# Beta cell primary cilia mediate somatostatin responsiveness via SSTR3

**DOI:** 10.1080/19382014.2023.2252855

**Published:** 2023-09-03

**Authors:** Samantha E. Adamson, Zipeng A. Li, Jing W. Hughes

**Affiliations:** Department of Medicine, Division of Endocrinology, Metabolism & Lipid Research, Washington University School of Medicine, St. Louis, USA

**Keywords:** beta cell, calcium, islet, primary cilia, somatostatin, SSTR3

## Abstract

Somatostatin is a paracrine modulator of insulin secretion and beta cell function with pleotropic effects on glucose homeostasis. The mechanism of somatostatin-mediated communication between delta and beta cells is not well-understood, which we address in this study via the ciliary somatostatin receptor 3 (SSTR3). Primary cilia are membrane organelles that act as signaling hubs in islets by virtue of their subcellular location and enrichment in signaling proteins such as G-protein coupled receptors (GPCRs). We show that SSTR3, a ciliary GPCR, mediates somatostatin suppression of insulin secretion in mouse islets. Quantitative analysis of calcium flux using a mouse model of genetically encoded beta cell-specific GCaMP6f calcium reporter shows that somatostatin signaling alters beta cell calcium flux after physiologic glucose stimulation, an effect that depends on endogenous SSTR3 expression and the presence of intact primary cilia on beta cells. Comparative *in vitro* studies using SSTR isoform antagonists demonstrate a role for SSTR3 in mediating somatostatin regulation of insulin secretion in mouse islets. Our findings support a model in which ciliary SSTR3 mediates a distinct pathway of delta-to-beta cell regulatory crosstalk and may serve as a target for paracrine modulation.

## Graphical abstract

**Figure:**
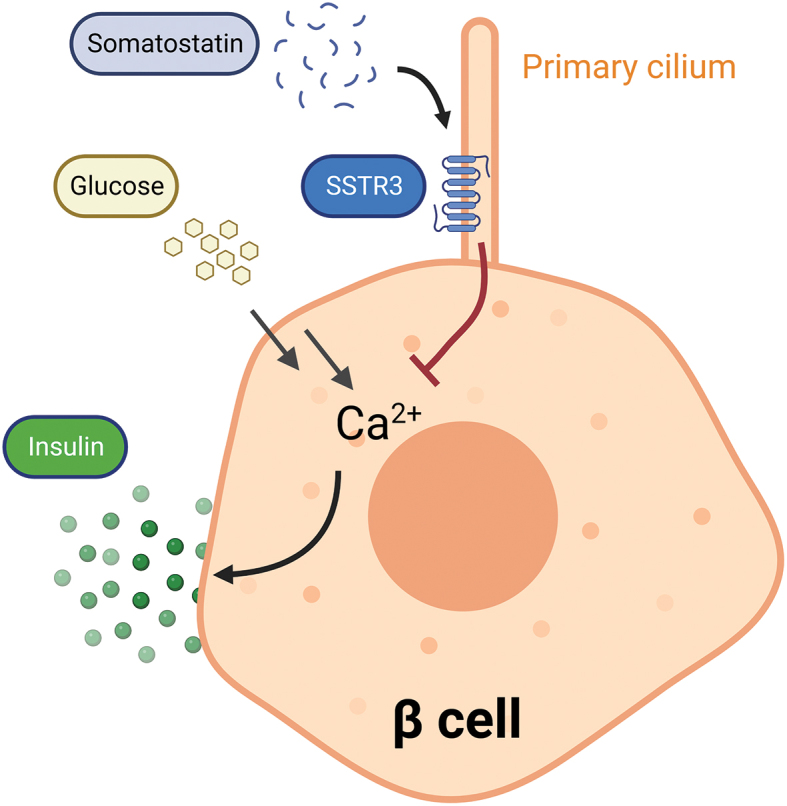

## Introduction

Proper blood glucose homeostasis requires integrated sensing and secretion of multiple hormones within pancreatic islets, including insulin (INS) from beta cells, glucagon (GCG) from alpha cells, and somatostatin (SST) from delta cells. Counterregulation of plasma glucose by insulin and glucagon is a well-characterized phenomenon in normal physiology, while dysregulation of these hormones leads to dysglycemia and is a hallmark of diabetes. The effect of somatostatin on plasma glucose is more nuanced because it simultaneously inhibits two opposing glucoregulatory hormones, glucagon and insulin.^1,2^ This gives rise to a complex clinical picture where somatostatin agonism and antagonism can produce overlapping effects. For example, nondiabetic patients treated with somatostatin analogs for growth hormone excess frequently develop hyperglycemia due to insulin suppression.^3,4^ Meanwhile, in insulin-dependent patients with diabetes, somatostatin and somatostatin analogs prevent DKA^5^ and improve both postprandial hyperglycemia^6–9^ and morning hyperglycemia^10^ by suppressing glucagon. Moreover, when glucagon secretion is inadequate, as in the setting of hypoglycemia in type 1 diabetes, somatostatin receptor antagonism improves counterregulatory responses to improve blood glucose stability.^11–13^ These varied observations likely stem from cell-specific effects of SST action, as well as spatially regulated somatostatin signaling within subcellular compartments. In a densely multi-cellular organ as the pancreatic islet, where a relatively small number of secreted molecules constitute the core paracrine pathways, the spatial positioning of receptors may provide a means to diversify the signaling interactions. Thus, a better understanding of the complexities of somatostatin biology, starting with its receptor distribution and signal transduction in specific islet cell subsets, could help minimize adverse effects on glycemia and lead to more targeted treatment modalities for diabetes.

The hormone somatostatin signals through five distinct G protein coupled receptors (GPCRs) named somatostatin receptor (SSTR) 1–5. While SSTR2 has been considered the predominant somatostatin receptor isoform in human islet alpha and beta cells, there is strong experimental evidence that SSTR3 also contributes to the paracrine signaling of somatostatin at least in beta cells.^14^ Studies have demonstrated SSTR3 expression at the transcriptome and protein levels across cell types in both mouse and human islets.^15–19^ SSTR3 is unique among all SSTRs in that its localization is restricted to the primary cilium, a slender cell-surface projection that senses extracellular cues and propagates those signals.^20^ SSTR3 is among a growing list of GPCRs found to be enriched in primary cilia, an organelle increasingly recognized as a specialized signaling hub.^21,22^ In beta cells, expression of SSTR3 is predominantly ciliary, suggesting a specific role for primary cilia in mediating somatostatin signaling.^23–25^

The primary cilium in pancreatic islet cells has emerged as a key locus of pancreas development and integration of hormone signaling.^26,27^ We^28^ and others^29^ have shown that cilia-deficient beta cells have defective glucose-stimulated insulin secretion. At the organism level, deletion of beta cell cilia in mice leads to increased fasting blood glucose levels and impaired glucose tolerance without obesity that was further exacerbated by high fat diet.^28^ In vitro, beta cell cilia-deficient islets demonstrate a loss of somatostatin-mediated insulin suppression,^28^ indicating a specific role for primary cilia in delta-to-beta cell crosstalk. In the present study, we go an important step further to show that SSTR3 is the ciliary receptor that mediates beta cell response to somatostatin, and that SSTR3 and cilia loss-of-function perturb first-phase and second-phase beta cell calcium flux, leading to defective paracrine SST regulation of insulin secretion. These results provide a mechanism by which primary cilia control intra-islet cell signaling and pinpoint SSTR3 as a specific ciliary target for modulating SST action in beta cells.

## Materials and methods

### Mice

Ins1-Cre mice from Jackson Laboratories (JAX #026801) were crossed to Ift88^fl/fl^ mice (JAX #022409) to generate a beta cell cilia-deficient mouse line (βCKO) as previously reported.^28^ βCKO was further crossed with GCaMP6f mice, generously provided by the laboratory of David Piston, producing a double transgenic line bearing both beta cell cilia knockout and beta cell calcium reporter. In parallel, a beta cell cilia reporter line was generated by crossing Ins1-Cre mice to SSTR3-GFP mice^24^ from the Yoder laboratory at University of Alabama at Birmingham. Mice were genotyped at weaning by a commercial vendor (Transnetyx). Mice were fed a standard rodent diet (PicoLab Mouse Diet 5053, 13.2% calories from fat) and used at 2 to 4 months of age for secretion and calcium experiments. Animals were maintained in accordance with Institutional Animal Care and Use Committee regulations at the Washington University School of Medicine.

### Islet isolation and culture

Islets were isolated from young adult mice using collagenase digestion with a modified Lacy protocol.^28,30^ Typical yield was 100 to 150 islets per mouse. Isolated islets were recovered overnight prior to experiments in a 37°C, 5% CO_2_ incubator in 10-cm plates with 10 mL islet media/plate (RPMI 1640 with 10% fetal bovine serum [FBS], penicillin – streptomycin, and 11 mmol/L glucose). For viral transfection, isolated islets were cultured overnight and then underwent partial digestion with Accutase (Innovative Cell Technologies) followed by treatment with 200 MOI Ad-mCherry-U6-scrmb-shRNA (#1781) or Ad-mCherry-U6-m-SSTR3-shRNA (Vector Biolabs). Ad-mCherry-U6-m-SSTR3-shRNA: 5’-CACCGCTTGTGCTACTTGCTCATTGCTCGAGCAATGAGCAAGTAGCACAAGC TTTTT-3.’ Islets were cultured on a microwell plate (AggreWell 800, Stemcell Technologies) with fresh media applied after 48 hours of viral treatment followed by an additional 48 hours in culture to allow for reaggregation.

### Immunocytochemistry

Isolated islets were washed with PBS and fixed with 4% paraformaldehyde (PFA) for 15 min and permeabilized with 0.3% Triton X-100 in PBS (PBST) for 10 min at room temperature. After incubation with blocking buffer (PBS with 10% normal goat serum) for 1 h at room temperature, islets were incubated overnight at 4°C with primary antibodies diluted in PBST. The next day, islets were washed, incubated with secondary antibodies for 1 h at room temperature, and washed again in PBST. DAPI (1:10,000) provided nuclear counterstain. Islets were mounted on glass slides with Prolong Gold Anti-fade (Thermo Fisher P36930). Islets were imaged using an inverted Zeiss LSM880 fluorescence microscope (Zeiss, Oberkochen, Germany). Primary antibodies and dilutions: 1:200 ARL13b (Abcam, Cat# ab136648), 1:400 ARL13b (Proteintech Cat# 17711–1-AP, RRID:AB_2060867), 1:400 acetylated tubulin (Sigma-Aldrich Cat# T7451, RRID:AB_609894), 1:400 polyglutamylated tubulin/GT335 (AdipoGen Cat# AG-20B-0020, RRID:AB_2490210), 1:100 SSTR3 (Thermo Fisher Scientific Cat# PA3–207, RRID:AB_10981488), 1:200 insulin (R&D Biosystems Cat# MAB1417, RRID:AB_2126533), secondary antibodies (Invitrogen) 1:500.

### Quantitative RT-PCR

Isolated mouse islets were lysed in 300 μL of RLT lysis buffer. RNA was purified with a Qiagen RNeasy mini kit, and cDNA was synthesized using a Thermo Fisher High-Capacity cDNA reverse transcription kit at 200 ng/20 μL. qPCR was performed on a 7900 Step One Plus RT-PCR machine (Applied Biosystems) using *Power*SYBR Green PCR Master Mix (Applied Biosystems). Changes in gene expression were quantified using 2^−∆∆CT^, and results were normalized to the housekeeping gene *Ppia*. Primer sequences for mouse *SSTR1–5* and *Ppia* mRNA are shown in Supplemental Table S1.

### Western blot

Isolated mouse islets were lysed in 20 μL of lysis buffer (20 mM Tris-HCl (pH 7.5), 150 mM NaCl, 1 mM Na_2_EDTA, 1 mM EGTA, 1% Triton, 2.5 mM sodium pyrophosphate, 1 mM beta-glycerophosphate, 1 mM Na_3_VO_4_, 1 µg/ml leupeptin, Cell Signaling, #9803) with protease inhibitor (Roche, #11836170001) for 30 min on ice with intermittent vortexing. Lysates were cleared of debris by centrifugation at 12,000 rpm for 30 min at 4°C. Supernatants were collected and protein content was determined via BCA Assay (ThermoFisher, #23225) according to the manufacturer’s instructions. Samples were mixed with 4X Laemmli Sample Buffer (Biorad, #1610747) with 1:10 2-mercaptoethanol (Sigma) and boiled at 95°C prior to SDS-PAGE analysis. Samples were loaded into 4–15% PAGE mini-PROTEAN TGX gel (Biorad, #4561084) and electrophoretically separated according to the manufacturer’s instructions (100 V for 60 min in Tris/Glycine/SDS Running Buffer, Biorad, #1610732). Separated proteins were transferred to nitrocellulose membrane 0.2 µm (Biorad, #1620112) for 90 min at 100 V in transfer buffer (Tris/Glycine 10% Methanol) at 4°C. Membrane was exposed to blocking buffer (TBS-tween 3% BSA) overnight at 4°C and then primary antibodies (1:1000 cyclophilin A, Proteintech, cat# 10720–1-AP, RRID:AB_2237516; 1:1000 GFP, Proteintech, cat# 50430–2-AP, RRID:AB_11042881) for 2 hours at room temperature. Membranes were washed with TBST and then exposed to secondary antibody (1:2000 anti-rabbit Igg HRP, Abcam, ab205718, RRID:AB_2819160) for 1 hour at room temperature followed by additional washes with TBST. Chemiluminescent substrate (SuperSignal West Pico PLUS, ThermoFisher, #34580) was applied per manufacturer instruction and then membranes were imaged using LI-COR Odyssey Fc imaging system.

### Static secretion assay

Islets were incubated in islet media with 400 nM SSTR3 antagonist MK-4256 (MedChemExpress, 1 mM stock solution in DMSO), 300 nM SSTR2 antagonist CYN-154806 (Tocris, 0.5 mM stock solution in 25 mM NaPO_4_ with 0.1% BSA), or control DMSO (Sigma) for 2 hours, and then equilibrated in Krebs-Ringer bicarbonate Hepes (KRBH) buffer (128.8 mmol/L NaCl, 4.8 mmol/L KCl, 1.2 mmol/L KH_2_PO_4_, 1.2 mmol/L MgSO_4_⋅7H_2_O, 2.5 mmol/L CaCl_2_, 20 mmol/L Hepes, 5 mmol/L NaHCO_3_, and 0.1% BSA [pH 7.4]) with or without continued antagonist treatment at 2 mmol/L glucose for 30 min at 37°C. Islets were then transitioned to 2, 8 or 11 mmol/L glucose in KRBH with or without antagonists for 1 h at 37°C in groups of 4 to 5 islets per tube. Initial experiments were performed with 11 mmol/L glucose, while a slightly lower and more physiologic level of stimulation with 8 mM rather than 11 mM glucose was used in later experiments including calcium imaging as it allows better resolution of beta cell Ca^2+^ responses near the glucose threshold.^31^ Islets were co-treated with 100 nM or 200 nM exogenous somatostatin peptide (Sigma, 40 µM stock solution in 25 mM NaPO_4_ with 0.1% BSA) during glucose stimulation. Supernatant containing secreted insulin was collected after 1 h, and islet hormone content was extracted overnight in acid-ethanol (1.5% 12 N HCl in 70% ethanol). Insulin concentrations were measured by a commercial mouse insulin enzyme-linked immunosorbent assay (ELISA) kit (CrystalChem, #90082), and secretion data were presented as a percentage of hormone content.

### Live-cell calcium imaging

Islets were equilibrated for 5 min in a climate-controlled microscope stage at 37°C, 5% CO_2_. All experiments took place in 2 mL buffer volume using MatTek glass bottom microwell dishes No. 1.5 (0.16 to 0.19 mm). Results were representative of three independent experiments. For each independent experiment islets were isolated from age and sex-matched mice; islets from both male and female mice were examined. Confocal imaging was conducted using a Zeiss 880 microscope with 488 nm laser excitation, and emission was detected at 500 to 570 nm using a spectral detector. Images were captured using a Zeiss Plan-Apochromat 20× objective and acquired at a frame size of 512 × 512 (135 × 135 µm^2^) with a pixel dwell time of 2.06 µs, resulting in a frame time of 0.633 s. The pinhole size was kept at 206.6 µm to allow for sufficient signal-to-noise ratio. Baseline time series images at 2 mM glucose were acquired for 320 s. Glucose concentration was then increased to 8 mM by manual pipetting followed by multiple imaging series of 320 s cycles. Purified somatostatin-28 peptide (Sigma) was used at working concentrations of 100–200 nM. Image series were concatenated, and intensity profiles for whole-islet regions of interest (ROIs) were generated for quantification (ImageJ). Data was normalized to low glucose (2 mM) condition.

Fourier transform analysis of calcium oscillations was performed on normalized calcium data with MatLab using the following script: n=length(‘data’); fs = 1.66666; dt = 1/fs; *t*=(0:*n*-1)/fs; f=(0:*n*-1)*(fs/n); y=fft(‘data’); power=(abs(y).^2)/n. Biologically relevant frequencies up to 0.2 s^−1^ were reported. The power spectrum represents the relative magnitudes of the frequency components that combine to form the signal and describes how the signal’s power varies with frequency.^32^ Frequencies with high power make up a greater proportion of the signal than frequencies with lower power. By comparing the power spectra of calcium signals in different conditions, we are able to understand when oscillations are slowed (higher power at lower frequencies) or quickened (higher power at higher frequencies). This analysis is especially useful in situations where there may be multiple relevant frequencies of oscillations such as in beta cell calcium flux.

### Statistics

Data are presented as mean ± SD unless otherwise indicated in figure legends. Statistical significance was analyzed by student t-test (two groups) or ANOVA (more than two groups) with Sidak’s post-hoc comparison test. Sidak’s post-hoc comparison test was used because it accounts for differences in variance among groups. Sample size and number of replicates for each experiment are indicated in figure legends.

## Results

### SSTR3 is expressed in islet cell cilia and required for paracrine regulation of insulin secretion

**Figure 1.**
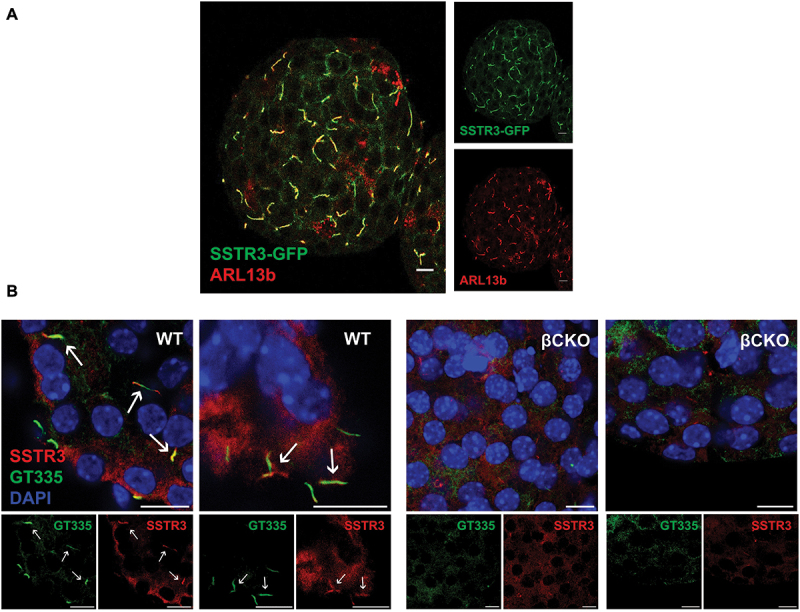
Colocalization of SSTR3 and primary cilia in pancreatic islets. a) Immunocytochemistry of cilia marker ARL13b (red) merged with SSTR3-GFP (green) in isolated islet from SSTR3-GFP mouse. Note the overlap of signal (yellow) between cilia marker and SSTR3, indicating expression of SSTR3 is restricted to primary cilia. b) Immunocytochemistry of isolated wildtype (WT: Ins1 Cre^+^ IFT88^wt/wt^) and beta cell cilia knockout (βCKO: Ins1 Cre^+^ IFT88^flfl^) islets with cilia marker GT335 (green), SSTR3 (red), and DAPI (blue) indicating nuclei. White arrows denote prominent cilia. Note overlap of cilia marker GT335 and SSTR3 in WT islets and absence of cilia in βCKO islets. Scale bar 10 µm.

**Figure 2.**
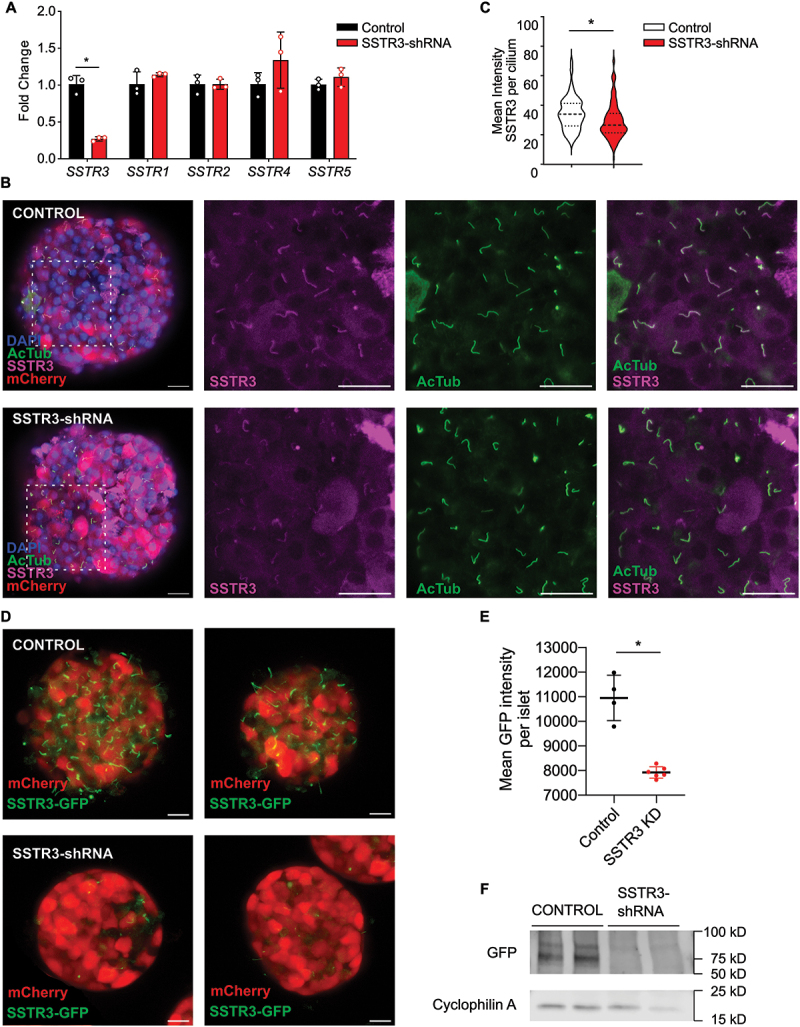
Specific knockdown of SSTR3 in mouse islets. a) Infection of mouse islets with SSTR3-shRNA (Ad-mCherry-U6-m-SSTR3-shRNA) results in a significant 70% decrease in *SSTR3* mRNA levels by RT-PCR compared to control (Ad-mCherry-U6-scrmb-shRNA) infected islets, while mRNA levels of *SSTR1*, *SSTR2*, *SSTR4*, and *SSTR5* are unchanged. *p = 0.0005 by Student’s t-test, data expressed as mean ± s.d. b) Immunocytochemistry confirms decreased SSTR3 (magenta) protein expression on primary cilia denoted by positive acetylated tubulin (AcTub, green) in SSTR3 KD islets compared to control. mCherry (red) shows similar transfection efficiency. DAPI (blue) indicating nuclei. Middle panels show single channel images of areas denoted by dotted white squares for SSTR3 (magenta) and AcTub (green). Right panels show merge of SSTR3 (magenta) and AcTub (green). c) Quantification of SSTR3 mean intensity per primary cilia ROI demonstrates a significant decrease in SSTR3 KD islets. N=control, 114 and SSTR3 KD, 148 primary cilia analyzed from 3 independent islets for each group. Data expressed as violin plot, *p < 0.0001 by Mann-Whitney test. d) Live islet images of islets isolated from SSTR3-GFP mice infected with control or SSTR3-shRNA demonstrate loss of SSTR3-GFP signal in SSTR3-shRNA treated islets. Scale bar 20 μm. e) Quantification of GFP mean intensity per islet demonstrates a significant decrease in SSTR3-shRNA treated islets. N=control, 4 and SSTR3-shRNA, 6 islets. Data expressed as mean ± s.d., *p = 0.006 by Student’s t-test with Welch’s correction. f) Loss of SSTR3 protein in whole islet lysates from SSTR3-shRNA treated SSTR3-GFP islets compared to control demonstrated by western blot probing for GFP. Cyclophilin A is loading control.

The expression of GPCR somatostatin receptor 3 (SSTR3) is restricted to the primary cilium.^23,24,33^ We confirmed that, in mouse islets, both the endogenous and GFP-tagged SSTR3 proteins localize to primary cilia as demonstrated by colocalization of SSTR3 with cilia markers ARL13b and GT335, while in beta cell cilia knockout (βCKO) islets, ciliary expression of SSTR3 is abolished on the majority of islet cells (Figure 1a–b), quantified in Supplemental Figure 1. To validate that non-ciliary signal from the SSTR3 antibody was nonspecific background signal from the antibody, co-staining of islets isolated from SSTR3-GFP mice with SSTR3 antibody demonstrated highly co-localized ciliary SSTR3 signal and background SSTR3 antibody signal that did not co-localize with SSTR3-GFP signal (Supplemental Figure 2). To determine the necessity and sufficiency of SSTR3 in mediating the ciliary-dependent somatostatin response, we utilized adenoviral vectors to knockdown SSTR3 with shRNA (SSTR3 KD). Wildtype islets transduced with SSTR3 shRNA (Ad-mCherry-U6-m-SSTR3-shRNA) adenoviral vector showed nearly 70% reduction of *SSTR3* mRNA expression by qPCR analysis compared to control (Ad-mCherry-U6-scrmb-shRNA), with no significant changes in mRNA expression of other isoforms of SSTR including *SSTR1*, *SSTR2*, *SSTR4*, and *SSTR5* (Figure 2a). Transfection efficiency denoted by mCherry expression is similar between control and SSTR3 KD islets (Supplemental Figure 3). SSTR3 KD islets maintain normal cilia morphology as labeled by the axonemal marker acetylated alpha tubulin (AcTub), and whereas control islets show robust co-localization of SSTR3 with AcTub, SSTR3 KD islets show diminished ciliary SSTR3 signal and reduced co-localization of SSTR3 with AcTub (Figure 2b). Mean intensity of SSTR3 signal per primary cilium is significantly decreased in SSTR3 KD compared to control (Figure 2c). To validate the SSTR3 knockdown model, islets isolated from SSTR3-GFP mice were treated with control or SSTR3-shRNA. SSTR3 knockdown SSTR3-GFP islets demonstrated significantly decreased GFP signal and loss of cilia morphology demonstrated by cilia-localized SSTR3-GFP in control islets (Figure 2d, e). Western blot of whole islet lysates from SSTR3-shRNA treated SSTR3-GFP islets demonstrated loss of SSTR3-GFP protein compared to control treated SSTR3-GFP islets (Figure 2f). As SSTR3 is the only somatostatin receptor known to be associated with beta cell primary cilia,^24^ SSTR3 KD represents a cilia-specific model of SSTR loss of function.

Colocalization of SSTR3 and primary cilia in pancreatic islets. a) Immunocytochemistry of isolated islet from SSTR3-GFP mouse shows overlap of SSTR3-GFP signal with slender cilia denoted by cilia marker ARL13b, indicating expression of SSTR3 is restricted to primary cilia. b) Immunocytochemistry of isolated wildtype (WT: Ins1 Cre+ IFT88wt/wt) islets shows slender cilia denoted by cilia marker GT335 overlap with SSTR3 signal with white arrows indicating prominent cilia; whereas, there is an absence of cilia in beta cell cilia knockout (βCKO: Ins1 Cre+ IFT88flfl) islets with no overlap of SSTR3 signal. Nuclei are denoted with DAPI. Scale bar 10 µm.

Specific knockdown of SSTR3 in mouse islets. a) Infection of mouse islets with SSTR3-shRNA (Ad-mCherry-U6-m-SSTR3-shRNA) results in a significant 70% decrease in SSTR3 mRNA levels by RT-PCR compared to control (Ad-mCherry-U6-scrmb-shRNA) infected islets, while mRNA levels of SSTR1, SSTR2, SSTR4, and SSTR5 are unchanged. *p=0.0005 by Student’s t-test, data expressed as mean ± s.d. b) Immunocytochemistry confirms decreased SSTR3 (magenta) protein expression on primary cilia denoted by positive acetylated tubulin (AcTub, green) compared to control. mCherry (red) shows similar transfection efficiency. DAPI (blue) indicating nuclei. Middle panels show single channel images of areas denoted by dotted white squares for SSTR3 (magenta) and AcTub (green). Right panels show merge of SSTR3 (magenta) and AcTub (green). c) Quantification of SSTR3 mean intensity per primary cilia ROI demonstrates a significant decrease in SSTR3 KD islets. N=control, 114 and SSTR3 KD, 148 primary cilia analysed from 3 independent islets for each group. Data expressed as violin plot, *p<0.0001 by Mann-Whitney test. d) Live islet images of islets isolated from SSTR3-GFP mice infected with control or SSTR3-shRNA demonstrate loss of SSTR3-GFP signal (green) in SSTR3-shRNA treated islets. mCherry (red) shows similar transfection efficiency. Scale bar 20 μm. e) Quantification of GFP mean intensity per islet demonstrates a significant decrease in SSTR3-shRNA treated islets compared to control. N=control, 4 and SSTR3 shRNA, 6 islets. Data expressed as mean ± s.d., *p=0.006 by Student’s t-test with Welch’s correction. f) Loss of SSTR3 protein in whole islet lysates from SSTR3-shRNA treated SSTR3-GFP islets compared to control demonstrated by western blot probing for GFP. Cyclophilin A is loading control.

**Figure 3.**
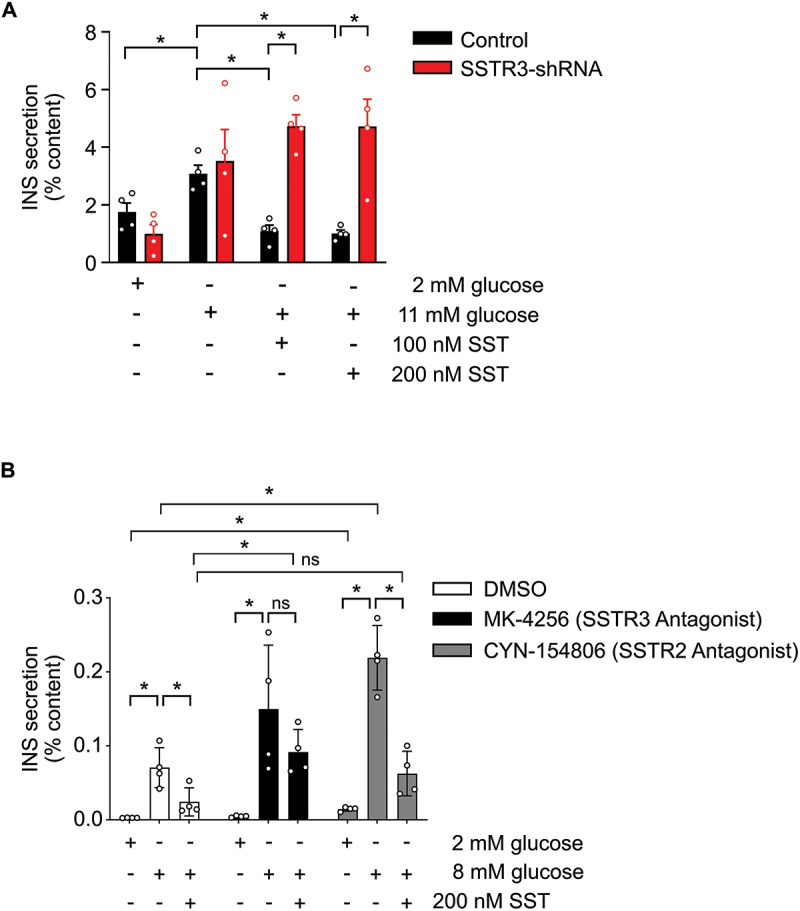
Static glucose-stimulated insulin secretion assay. a) Insulin secretion of control and SSTR3-shRNA treated wildtype mouse islets exposed to glucose for 1 hour with and without SST-28. 1-way ANOVA with Sidak’s multiple comparison test, *p < 0.05, data expressed as mean ± s.e.m., *n* = 4 replicates. b) Insulin secretion of wildtype mouse islets pretreated for 2 hr with 400 nM MK-4256 (SSTR3 antagonist), 300 nM CYN-154806 (SSTR2 antagonist), or control DMSO exposed to glucose for 1.5 hours with and without SST-14. 1-way ANOVA with Sidak’s multiple comparison test, *p < 0.02, data expressed as mean ± s.d., *n* = 4 replicates.

Static glucose stimulated insulin secretion assay. a) Control islets have significantly increased insulin secretion when exposed to 11 mM glucose compared to 2 mM glucose, and insulin secretion is significantly decreased by the presence of either 100 nM or 200 nM SST-28 with 11 mM glucose. SSTR3 knockdown islets have significantly increased insulin secretion when exposed to 11 mM glucose compared to 2 mM glucose, similar to control islets, but insulin secretion is not significantly decreased by the presence of either 100 nM or 200 nM SST-28 with 11 mM glucose and is significantly higher than control islets exposed to 100 nM or 200 nM SST-28 with 11 mM glucose. 1-way ANOVA with Sidak’s multiple comparison test, *p<0.05, data expressed as mean ± s.e.m., n=4 replicates. b) Wildtype mouse islets pretreated for 2 hr with 400 nM MK-4256 (SSTR3 antagonist), 300 nM CYN-154806 (SSTR2 antagonist), or control DMSO exposed to glucose for 1.5 hours with and without SST-14. Both control DMSO-treated islets and islets pretreated with CYN-154806 have significantly increased insulin secretion when exposed to 8 mM glucose compared to 2 mM glucose, and insulin secretion is significantly decreased by the presence of 200 nM SST-14 with 8 mM glucose. Islets pretreated with MK-4256 have significantly increased insulin secretion when exposed to 8 mM glucose compared to 2 mM glucose, similar to DMSO-treated islets, but insulin secretion is not significantly decreased by the presence of 200 nM SST-14 with 8 mM glucose and is significantly higher than DMSO-treated islets exposed to 100 nM SST-14 with 8 mM glucose. Islets pretreated with CYN-154806 have significantly higher insulin secretion when exposed to 2 mM glucose or 8 mM glucose compared to DMSO-treated islets. 1-way ANOVA with Sidak’s multiple comparison test, *p<0.02, data expressed as mean ± s.d., n=4 replicates.

We tested the effect of SSTR3 KD on glucose-stimulated insulin secretion (GSIS). Both control and SSTR3 KD mouse islets exhibited normal GSIS from 2 to 11 mM glucose, indicating that SSTR3 is not required for normal GSIS. However, SSTR3 KD mouse islets exhibited a select defect in GSIS regulation by somatostatin. Whereas GSIS was normally suppressed in control islets by both 100 nM and 200 nM somatostatin, SSTR3-KD islets displayed no suppression of GSIS at either concentration of somatostatin and continued to secrete insulin at maximal levels (Figure 3a). As SSTR2 is thought to be a predominant SSTR in islets whereas SSTR3 is the uniquely ciliary SSTR, we sought to determine the relative contribution of SSTR3 and SSTR2 in mediating paracrine SST suppression of insulin secretion in beta cells. Isolated islets were treated for 2 hours with the SSTR3 antagonist MK-4256, the SSTR2 antagonist CYN-154806, or control DMSO, and then subjected to GSIS. Whereas SSTR3 antagonist treatment diminished SST suppression of GSIS, DMSO and SSTR2 antagonist treatment did not affect SST action on insulin secretion (Figure 3b). These results demonstrate that SSTR3 signaling is part of the physiologic paracrine pathway of delta cell inhibition of beta cell insulin secretion. Notably, treatment of islets with the SSTR2 antagonist CYN-154806 significantly increased the amount of insulin secreted in both 2 mM glucose and 8 mM glucose compared to DMSO-treated islets, indicating that SSTR2 signaling may be important for tonic feedback to beta cells.

### Primary cilia regulate intracellular calcium responses to glucose and somatostatin

Because SSTR3 expression is localized to primary cilia, we hypothesized that loss of the ciliary structure and therefore abolishment of SSTR3 localization would disrupt SST-mediated signaling in beta cells leading to calcium flux abnormalities. Previously we have used cell-permeant Fluo4 dye to demonstrate diminished calcium responses in beta cell cilia knockout islets.^28^ However, this method has technical limitations including variability of dye loading and dye penetrance into cells within the islet core. To overcome these limitations, we generated double transgenic mice in which the calcium reporter GCaMP6f^34^ is expressed in beta cells via INS1-Cre, while beta cell cilia are ablated by floxed deletion of IFT88, a intraflagellar transport protein essential for cilia assembly and maintenance. Immunocytochemistry of islets isolated from knockout mice (βCKO-GCaMP: Ins1 Cre^+/+^ Gcamp6^mut^ IFT88^fl/fl^) confirmed specific deletion of primary cilia in beta cells, in contrast to abundant cilia expression in wildtype islet beta cells (WT-GCaMP: Ins1 Cre^+/+^ Gcamp6^mut^ IFT88^wt/wt^) (Supplemental Figure 4a). While the fluorescent GCaMP6 signal is not seen in the stained image on account of islets being maintained in low glucose prior to fixation and staining, we detect strong and reliably glucose-sensitive green GCaMP6 fluorescence in all live imaging studies (Supplemental Figure 4b).

**Figure 4.**
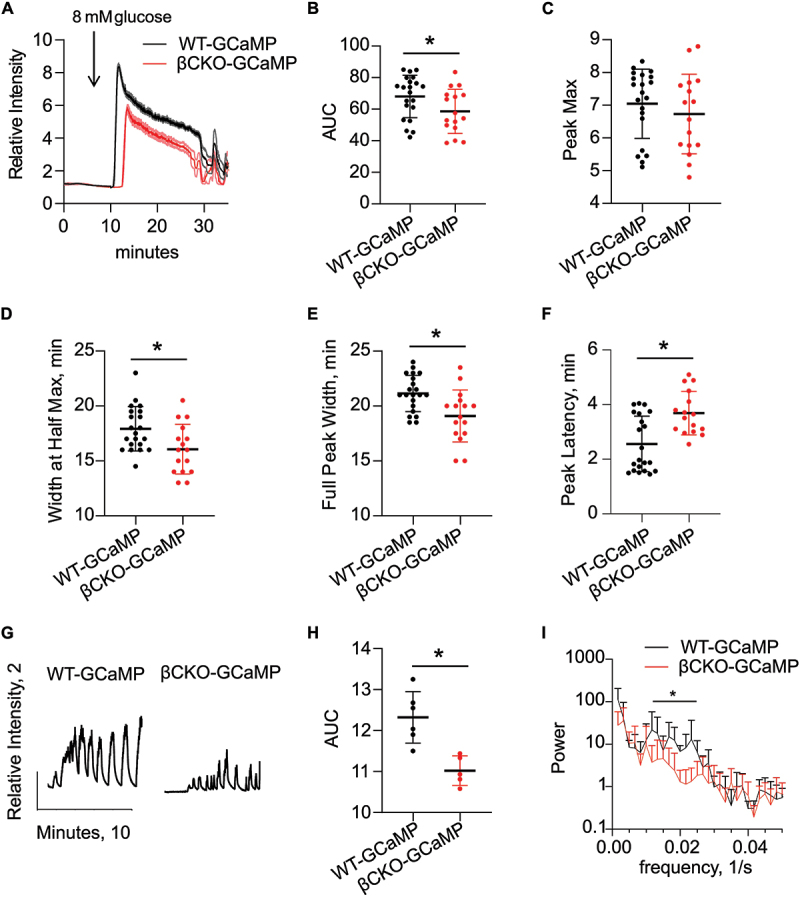
Primary cilia are required for normal glucose-stimulated first- and second-phase calcium flux in beta cells. a) Representative normalized calcium traces from WT-GCaMP (*n* = 7) and βCKO-GCaMP (*n* = 4) islets in 2 mM glucose treated with 8 mM glucose at time indicated by arrow. Data expressed as mean (WT-GCaMP, black line; βCKO-GCaMP, red line) ± s.e.m. (WT-GCaMP, gray line; βCKO-GCaMP, light red line). b) Quantification of area under the curve (AUC) of initial calcium peak. C) Initial calcium peak maxima. d) Initial calcium peak width at half max in minutes. e) Initial calcium peak width at base. f) Time to onset or peak latency of calcium signal after addition of high glucose. b-f) Each point represents one islet, WT-GCaMP (*n* = 21) and βCKO-GCaMP (*n* = 16), from 3 separate experiments, data shown as mean ± s.d., *p < 0.05 by Student’s t-test. g) Representative normalized calcium traces of second-phase calcium oscillations in WT-GCaMP and βCKO-GCaMP islets 20 minutes after treatment with 8 mM glucose. h) Quantification of area under the curve (AUC) of second-phase calcium oscillations. Each point represents one islet, *p = 0.003 by Student’s t-test. I) Fourier transform analysis of second-phase calcium oscillations of WT-GCaMP (*n* = 6) and βCKO-GCaMP (*n* = 8) islets. 2-way ANOVA with Sidak’s multiple comparison test, *p < 0.004, data expressed as mean ± s.d.

Primary cilia are required for normal glucose-stimulated first- and second-phase calcium flux in beta cells. a) Representative normalized calcium traces from WT-GCaMP (n=7) and βCKO-GCaMP (n=4) islets in 2 mM glucose treated with 8 mM glucose at time indicated by arrow demonstrate first-phase calcium peak. Data expressed as mean (WT-GCaMP, black line; βCKO-GCaMP, red line) ± s.e.m. (WT-GCaMP, grey line; βCKO-GCaMP, light red line). b) Area under the curve (AUC) of initial calcium peak is significantly decreased in βCKO-GCaMP islets compared to WT-GCaMP. c) Initial calcium peak maxima is not significantly different between WT-GCaMP and βCKO-GCaMP islets. Initial calcium peak width at half max (d) and initial calcium peak width at base (e) in minutes is significantly decreased in βCKO-GCaMP islets compared to WT-GCaMP. f) Time to onset or peak latency of calcium signal after addition of high glucose is significantly increased in βCKO-GCaMP islets compared to WT-GCaMP. b-f) Each point represents one islet, WT-GCaMP (n=21) and βCKO-GCaMP (n=16), from 3 separate experiments, data shown as mean ± s.d., *p<0.05 by Student’s t-test. g) Representative normalized calcium traces of second-phase calcium oscillations in WT-GCaMP and βCKO-GCaMP islets 20 minutes after treatment with 8 mM glucose demonstrate robust calcium oscillations in WT-GCaMP while βCKO-GCaMP oscillations are slower with smaller amplitude. H) AUC of second-phase calcium oscillations is significantly decreased in βCKO-GCaMP islets compared to WT-GCaMP. Each point represents one islet, *p=0.003 by Student’s t-test. I) Fourier transform analysis of second-phase calcium oscillations of WT-GCaMP (n=6) and βCKO-GCaMP (n=8) islets demonstrates significantly slower oscillations in βCKO-GCaMP islets compared to WT-GCaMP. 2-way ANOVA with Sidak’s multiple comparison test, *p<0.004, data expressed as mean ± s.d.

For analysis of calcium response, we quantified both the kinetics and magnitude of calcium peaks in both WT-GCaMP and βCKO-GCaMP islets. WT islets showed a robust rise in intracellular calcium in response to 8 mM physiologic stimulatory glucose. This response was both delayed and diminished in beta cell cilia-deficient islets (Figure 4a). The area under the curve (AUC) for the initial calcium peak was significantly lower in βCKO-GCaMP islets compared to WT (Figure 4b). Peak maximum was not significantly different between WT-GCaMP and βCKO-GCaMP islets (Figure 4c), but peak width was significantly decreased in βCKO-GCaMP islets (Figure 4d–e). Peak latency was increased by approximately 1.1 ± 0.2 minutes in βCKO-GCaMP islets (Figure 4f). Thus, these results show that primary cilia regulate both the onset and magnitude of the initial beta cell response to glucose.

While the initial calcium response peak corresponds to first-phase insulin secretion, second phase calcium oscillations are associated with the longer second-phase of insulin secretion, which is responsible for the majority of total insulin secretion and is well-characterized as responsive to paracrine modulators.^2,35–37^ WT-GCaMP islets showed robust second-phase calcium oscillations after 8 mM glucose addition in all islets examined, whereas in βCKO-GCaMP islets, fewer entered second-phase calcium oscillations, and the amplitude and AUC of those oscillations were significantly diminished compared to WT-GCaMP islets (Figure 4g–h). Fourier transform analysis of second-phase calcium oscillations revealed that WT islets had significantly more oscillations in frequency range of 0.01 to 0.02 oscillations per second compared to βCKO-GCaMP islets, suggesting that when βCKO-GCaMP islets are able to sustain second-phase calcium oscillations, these oscillations tend to be slightly slower compared to WT islets. These data indicate that primary cilia are important for both first- and second-phase calcium signaling in beta cells in response to glucose. This provides a mechanistic explanation for the decreased insulin secretion in βCKO-GCaMP islets and for our previous observation of impaired glucose homeostasis in beta cell cilia knockout animals.^28^

### Primary cilia are required for regulation of beta cell calcium flux by somatostatin

**Figure 5.**
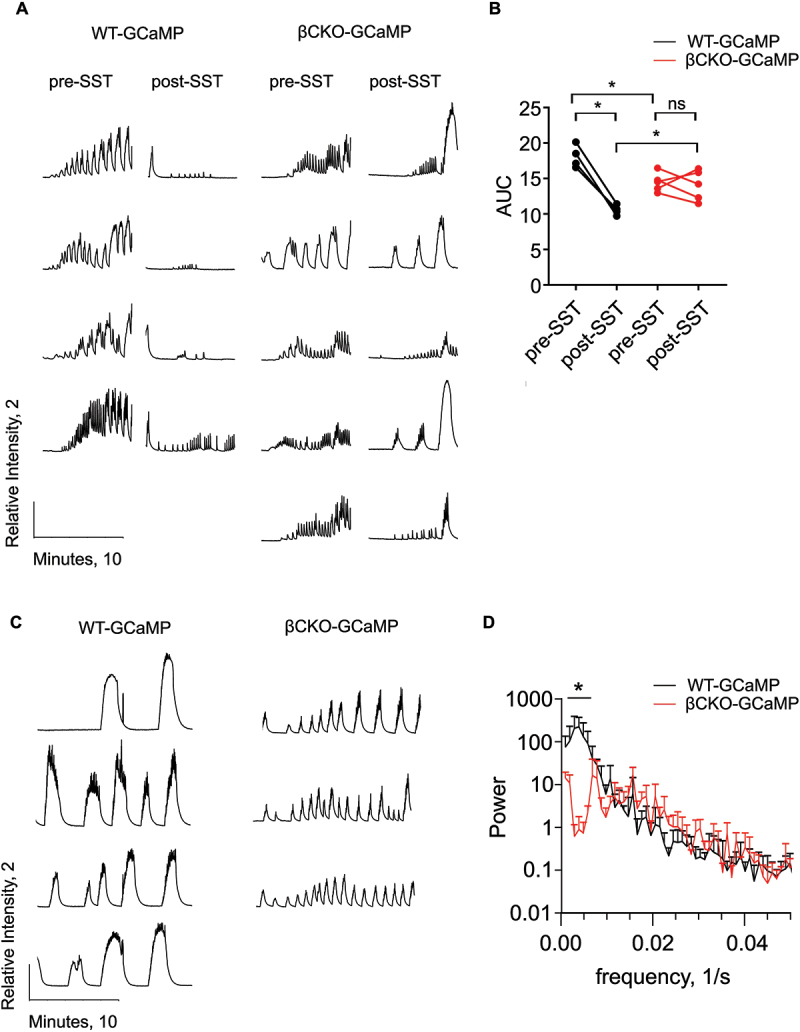
Primary cilia are required for SST-mediated changes in calcium flux in beta cells. a) Individual traces of normalized calcium intensity over time for WT-GCaMP and βCKO-GCaMP islets in high glucose (8 mM) condition pre and post addition of 200 nM SST. Islets were equilibrated in low glucose (2 mM) condition. Pre-SST data were captured 20 minutes after addition of high glucose (8 mM). Post-SST data were captured 5 minutes after the addition of 200 nM SST. Scale bar indicates normalized intensity of 2 and time of 10 minutes. b) Area under the curve of normalized calcium intensity plots. Each set of linked points represents one islet pre- and post-somatostatin. 1-way ANOVA with Sidak’s multiple comparison test, *p < 0.02. c) Individual traces of normalized calcium intensity over time for WT-GCaMP and βCKO-GCaMP islets 20 minutes after simultaneous treatment with 8 mM glucose and 200 nM SST. Scale bar indicates normalized intensity of 2 and time of 10 minutes. d) Fourier transform analysis of second-phase calcium oscillations of WT-GCaMP (*n* = 4) and βCKO-GCaMP (*n* = 3) islets. 2-way ANOVA with Sidak’s multiple comparison test, *p < 0.0005, data expressed as mean ± s.d.

Because loss of primary cilia disrupts expression and localization of the SST receptor SSTR3, we hypothesized that beta cell cilia-deficient islets would lack responsivity to SST in the suppression of glucose-induced calcium transients. We treated WT-GCaMP and βCKO-GCaMP islets with 8 mM glucose and observed second-phase oscillations 20 minutes after 8 mM glucose; islets were then treated with somatostatin, and changes in intracellular calcium were measured. We observed significantly reduced second-phase calcium oscillations as measured by AUC in βCKO-GCaMP islets compared to WT-GCaMP (Figure 5a–b). Somatostatin caused rapid and marked suppression of calcium oscillations in WT-GCaMP islets (Figure 5a–b). In contrast, βCKO-GCaMP islets responded to somatostatin to a lesser degree and did not significantly reduce calcium flux post-SST (Figure 5a–b). We also assessed second-phase calcium oscillations in islets treated simultaneously with 8 mM glucose and somatostatin. Second-phase calcium oscillations 20 minutes after simultaneous treatment with glucose and somatostatin in WT-GCaMP islets were notably broader and slower compared to βCKO-GCaMP islets (Figure 5c), as quantified by Fourier transform analysis which demonstrated that WT-GCaMP islets had significantly higher representation of low frequency oscillations (Figure 5d). The ability of βCKO-GCaMP islets to maintain higher frequency second-phase calcium oscillations in spite of somatostatin treatment is consistent with the observation that cilia-deficient islets cannot suppress insulin secretion in response to somatostatin. Collectively, these data demonstrate that primary cilia and specifically ciliary SSTR3 mediate the somatostatin-induced paracrine effects on calcium oscillation and insulin secretion in beta cells.

### Somatostatin signaling via SSTR3 controls beta cell calcium dynamics

**Figure 6.**
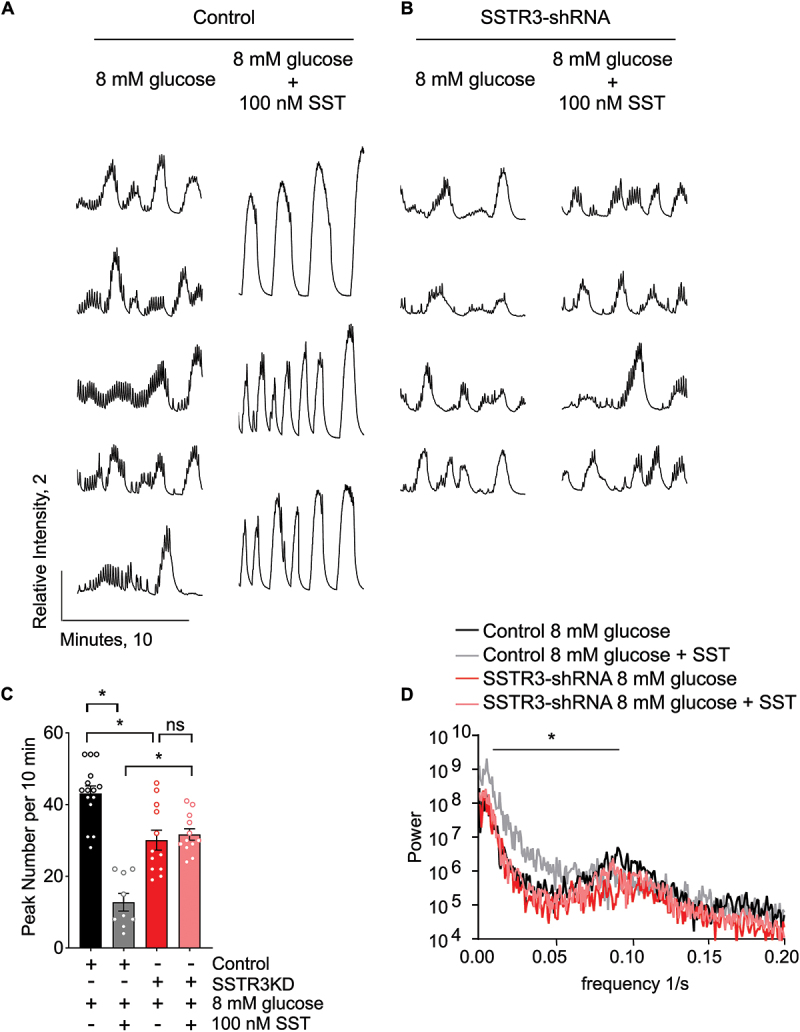
SSTR3 is required for slowing of calcium oscillations in response to SST. a,b) Individual traces of normalized calcium intensity over time for WT control and SSTR3-shRNA islets 30 minutes after treatment with high glucose (8 mM) with or without simultaneous addition of 100 nM somatostatin (SST). Scale bar indicates normalized intensity of 2 and time of 10 minutes. c) Quantification of calcium oscillations by 3 different blinded observers. 1-way ANOVA with Sidak’s multiple comparison test, *p < 0.0005, data expressed as mean ± s.e.m. d) Fourier transform analysis of calcium oscillations. 2-way ANOVA with Sidak’s multiple comparison test, *p < 0.0001, data expressed as mean. For c and d, *n* = 5, 3, 4, 4 for control 8 mM glucose, control 8 mM glucose +100 nM SST, SSTR3-shRNA 8 mM glucose, SSTR3-shRNA 8 mM glucose +100 nM SST, respectively.

To further test the requirement for endogenous SSTR3 in mediating beta cell calcium responses to SST, WT-GCaMP islets were subjected to control or SSTR3 knockdown by viral shRNA prior to calcium imaging. Islets were equilibrated to low glucose (2 mM) and then exposed to stimulatory glucose (8 mM) alone or simultaneous 8 mM glucose and 100 nM somatostatin in static incubation. Cytosolic Ca^2+^ was continuously monitored for up to 50 minutes post-stimulation on a climate-controlled confocal microscope. Control islets exhibited normal biphasic calcium responses including synchronous second-phase oscillations approximately 30 minutes after glucose stimulation. Control islets treated with 8 mM glucose demonstrated fast and sustained second-phase calcium oscillations, which were slowed down by co-treatment with 100 nM somatostatin (Figure 6a). This SST-dependent inhibition was lost in SSTR3 knockdown islets, which had similarly fast oscillations when treated with 8 mM glucose or with 8 mM glucose and 100 nM somatostatin (Figure 6b). Quantification of peak number (Figure 6c) as well as Fourier transform analysis of second-phase calcium oscillations (Figure 6d) verified that simultaneous addition of glucose and somatostatin results in slower second-phase oscillations than addition of glucose alone in control islets, but there is no difference in SSTR3 knockdown islets. These data indicate that SSTR3 is a critical mediator of slowing of calcium oscillations by SST but may not be required for silencing of calcium oscillations already underway.

SSTR3 is required for slowing of calcium oscillations in response to SST. a, b) Individual traces of normalized calcium intensity over time for WT control and SSTR3-shRNA islets 30 minutes after treatment with high glucose (8 mM) with or without simultaneous addition of 100 nM somatostatin (SST). Control islets treated with 8 mM glucose have robust and rapid oscillations while simultaneous treatment with 100 nM SST and 8 mM glucose causes oscillations to become much slower. SSTR3 knockdown islets have rapid oscillations after treatment with 8 mM glucose that are not different compared to oscillations after simultaneous treatment with 100 nM SST and 8 mM glucose. Scale bar indicates normalized intensity of 2 and time of 10 minutes. c) Quantification of calcium oscillations by 3 different blinded observers demonstrates significantly fewer peaks in control islets treated with SST and glucose compared to glucose alone, while there is no difference between SSTR3 knockdown islets treated with SST and glucose compared to glucose alone. 1-way ANOVA with Sidak’s multiple comparison test, *p<0.0005, data expressed as mean ± s.e.m. d) Fourier transform analysis of calcium oscillations shows significantly slower oscillations in control islets treated with SST and glucose compared to glucose alone or SSTR3 knockdown islets treated with SST and glucose or glucose alone. 2-way ANOVA with Sidak’s multiple comparison test, *p<0.0001, data expressed as mean. For C and D, n=5, 3, 4, 4 for control 8 mM glucose, control 8 mM glucose + 100 nM SST, SSTR3-shRNA 8 mM glucose, SSTR3-shRNA 8 mM glucose + 100 nM SST, respectively.

**Figure 7.**
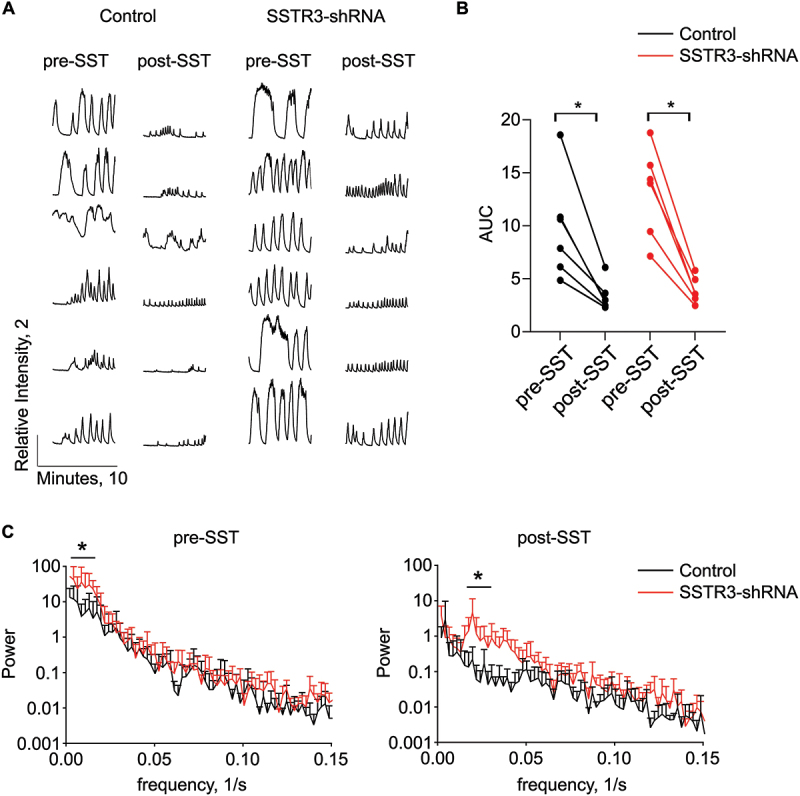
In response to SST, SSTR3 knockdown islets have calcium oscillations with suppressed amplitude that are more rapid. a) Individual traces of normalized calcium intensity over time for control and SSTR3-knockdown islets 20 minutes after treatment with 8 mM glucose (pre-SST) and then 7 minutes after addition of 200 nM somatostatin (post-SST). Scale bar indicates normalized intensity of 2 and time of 10 minutes. b) Area under the curve of normalized calcium intensity plots. Each set of linked points represents one islet pre- and post-somatostatin. 1-way ANOVA with Sidak’s multiple comparison test, *p < 0.02. c) Fourier transform analysis of calcium oscillations pre- and post-SST, *n* = 6 for each group. 2-way ANOVA with Sidak’s multiple comparison test, *p < 0.0001, data expressed as mean ± s.D.

To specifically test the role of paracrine SST during second-phase calcium oscillations, we performed calcium imaging using delayed treatment of SST. The addition of somatostatin after the onset of second-phase glucose-induced calcium oscillations resulted in silencing of subsequent calcium flux in control treated islets, which was expected (Figure 7a–b). In contrast, while SSTR3 knockdown islets retained the ability to suppress calcium flux in response to second-phase somatostatin addition (Figure 7a–b), careful analysis revealed that SSTR3 knockdown islets experienced significantly slower oscillations compared to control islets prior to the addition of somatostatin and significantly faster oscillations after the addition of somatostatin (Figure 7c). Calcium findings are compared between the genetic knockout of primary cilia in beta cells and shRNA knockdown of SSTR3 in islets in Supplemental Table S2. Thus, these data support a context-dependent role for SSTR3 in mediating somatostatin induced changes in calcium flux, depending on when the beta cell sees paracrine SST during its glucose-stimulated response.

In response to SST, SSTR3 knockdown islets have calcium oscillations with suppressed amplitude that are more rapid. a) Individual traces of normalized calcium intensity over time for control and SSTR3-knockdown islets 20 minutes after treatment with 8 mM glucose (pre-SST) and then 7 minutes after addition of 200 nM somatostatin (post-SST). Pre-SST oscillations are robust and rapid in both control and SSTR3 knockdown islets. Post-SST oscillations are of lower amplitude in both control and SSTR3 knockdown islets; however, oscillations appear more rapid in SSTR3 knockdown islets compared to control. Scale bar indicates normalized intensity of 2 and time of 10 minutes. b) Area under the curve of normalized calcium intensity plots demonstrates significantly decreased calcium flux post-SST compared to pre-SST in both control and SSTR3 knockdown islets. Each set of linked points represents one islet pre- and post-somatostatin. 1-way ANOVA with Sidak’s multiple comparison test, *p<0.02. c) Fourier transform analysis of calcium oscillations pre- and post-SST shows that pre-SST oscillations are significantly slower in SSTR3 knockdown islets while post-SST oscillations are significantly faster in SSTR3 knockdown islets compared to control. N=6 for each group. 2-way ANOVA with Sidak’s multiple comparison test, *p<0.0001, data expressed as mean ± s.d.

## Discussion

Communication among beta, alpha, and delta cells within the three-dimensional structure of the islet is essential for normal physiological responses,^38^ given that islet cells lose functionality when dispersed from intact islets into single cells.^39–43^ Our data implicate primary cilia as important mediators of intercellular communication between pancreatic delta and beta islet cells. We demonstrate that SSTR3 is expressed on beta cell primary cilia and mediates somatostatin signaling from delta to beta cells to modulate insulin secretion.

Primary cilia-deficient beta cells fail to suppress both calcium flux and insulin secretion in response to somatostatin, a phenotype partially recapitulated by SSTR3 knockdown, suggesting that cilia-localized SSTR3 is important for delta-beta crosstalk. While SSTR2 has long been thought to be the predominant mediator of somatostatin-induced suppression of insulin secretion, our data shows that SSTR3 mediates this regulation in beta cells and does so via a unique ciliary pathway. However, we observed that while islets treated with a specific SSTR2 antagonist suppressed glucose-stimulated insulin secretion normally in the presence of SST, these islets had significantly increased insulin secretion in both 2 mM glucose and 8 mM glucose compared to DMSO-treated islets, demonstrating a role for SSTR2 signaling in the regulation beta cell activity in response to endogenous SST tone. It is likely that both SSTR2 and SSTR3 contribute to delta-to-beta cell regulatory crosstalk via different aspects of SST-mediated effects such as endogenous SST tone versus dynamic response to SST in changing glucose concentrations, but we show that SSTR3 signaling is localized to primary cilia. We validated our genetic knockdown results using pharmacologic agents, via selective SSTR3 antagonism which as a therapeutic approach has shown promise as a treatment for type 2 diabetes by boosting insulin secretion in preclinical models and in human islets *ex vivo*.^44^ While systemic use of SSTR3 modulators is currently limited by extrapancreatic side effects, including cardiac QTc prolongation in dogs,^45^ our findings show that beta cell- and cilia-specific SSTR3 modulation may be a viable approach for restoring insulin secretion through islet cell crosstalk.

Several limitations were present in our study stemming from both the experimental system and available reagents. One, we relied on exogenously supplemented, recombinant SST peptides to test the effect of SST on beta cell activity, while endogenous contributions by delta cells were not accounted for. Two, immunocytochemical images obtained with polyclonal antibodies against SSTR3 contained heavy background signal which is likely nonspecific, as a complementary model using SSTR3-GFP expressing islets show less non-ciliary GFP expression, and SSTR3 shRNA in both native and SSTR3-GFP reporter islets diminish ciliary and overall GFP signal, respectively. As an additional validation, ciliary specificity of SSTR3 expression of islets was also reported in the original manuscript that described the development of the SSTR3-GFP mouse strain.^24^

Another limitation was the approach using virally mediated SSTR3 knockdown in isolated islets, which, unlike the cre-mediated gene deletion and calcium reporter mouse models we employed, was not beta cell-specific and therefore likely affected more than one islet cell type. Disrupted SSTR3 expression on alpha and delta cells could have had feedback effects on beta cell SSTR3 signaling, thus future experiments might study the role of SSTR3 in beta cells in isolation, e.g. MIN6 cells, or separately address the effect of glucagon and somatostatin perturbation by loss of SSTR3 in alpha and delta cells in whole islets. Reassuringly, in our experimental model, loss of SSTR3 on islet cells overall produced a strong beta cell phenotype, which likely resulted from direct beta cell-specific effects of SSTR3 loss, leading to an inability to suppress insulin secretion in response to somatostatin.

The present study offers an important technical advance over existing data, namely the development of the double transgenic beta cell-specific cilia knockout and GCaMP6f calcium reporter mouse model. We previously reported calcium flux perturbations in beta cell cilia knockout (βCKO) islets^28^ using cell-permeant Fluo4 calcium dye, whereas our current approach using a genetically encoded calcium indicator is cell type-specific and optimizes for calcium affinity and response speed. To visualize calcium oscillatory behavior over the entire duration of the beta cell glucose response, we chose a closed imaging system instead of perifusion in order to recapitulate the calcium dynamics occurring in our static islet hormone secretion assay. Both first- and second-phase calcium oscillations are observed and quantifiable in this setting, showing that static incubation allows production of physiologic beta cell responses to glucose and to paracrine factors. Future studies may address potential differences in static versus dynamically perifused islet experimental setups, and whether this impacts calcium and insulin secretion quantitation.

Calcium oscillations are driven by oscillations in membrane potential which involve multiple classes of ion channels.^46^ A key finding is that fewer βCKO islets enter into sustained Ca^2+^ oscillations during physiologic 8 mM glucose stimulation, which may result from a shift in beta cell glucose sensitivity, or an inherent defect in their Ca^2+^ oscillation apparatus, or both. Experiments using higher glucose concentrations may help clarify these issues, as well as studies of cilia loss effects on beta cell metabolism versus function of calcium or potassium channels. Our experiments showed that addition of somatostatin simultaneously with glucose caused a slowing and broadening of calcium oscillatory signals as opposed to silencing calcium flux, suggesting a modulatory rather than strictly inhibitory effect. Somatostatin regulation of calcium signaling on the plasma membrane occurs through multiple mechanisms including inhibition of L-type voltage gated calcium channels,^47–50^ activation of inwardly rectifying (GIRK) potassium channels,^51,52^ and Na^+^/K^+^ ATPases.^53^ Whether any of these channels play a role in cilia-mediated calcium regulation is unknown in beta cells. L-type calcium channels have been identified in primary cilia,^54,55^ thus it is possible that SSTR3 may physically or functionally interact with a ciliary voltage-gated calcium channel, and future studies examining ciliary calcium dynamics in the beta cell might find a connection with cytoplasmic calcium dynamics.^56^ Additionally, voltage-gated calcium channels are abundantly expressed on the plasma membrane,^57,58^ so our findings are equally compatible with the possibility that SSTR3 relays a signal from primary cilia to the rest of the cell surface to regulate global calcium entry. Other ciliary-specific channels such as the transient receptor potential cation channel polycystin 2^54,59,60^ may play a role in regulating beta cell calcium flux, and SSTR3-activated cAMP signaling, which was not examined in this study may also regulate intracellular calcium dynamics.^61,62^

In summary, our data reveals a strong role for the beta cell primary cilium as a hub of somatostatin signaling in islets, necessary for the physiologic crosstalk between delta and beta cells. Beta cell primary cilia are required for transducing glucose-stimulated first-phase calcium flux, second-phase calcium oscillations, and for suppression of calcium responses by somatostatin. Localization of key membrane proteins such as GPCRs to primary cilia creates a unique microdomain enriched in paracrine signaling capabilities, which, in beta cells, modulate insulin secretion in response to intra-islet somatostatin. Our findings reveal SSTR3 as a ciliary GPCR that mediates somatostatin action on beta cells. Thus, targeting SSTR3 and potential associated ciliary signaling pathways may be a useful strategy to modulate beta cell sensitivity to extracellular cues.

## Supplementary Material

Supplemental Material
