# Supplementary material for: Beta cell primary cilia mediate somatostatin responsiveness via SSTR3
Source: Islets. 2023 Sep 3;15(1):2252855. doi: 10.1080/19382014.2023.2252855 (PMC10478741; doi:10.1080/19382014.2023.2252855)
Supplement: Supplemental Material [file KISL_A_2252855_SM3299.zip › Supplemental Table 1.docx]

Supplemental Table 1. RT-PCR Primer Sequences:

| Gene | Forward primer sequence: | Reverse primer sequence |
| --- | --- | --- |
| Sstr1 | CAT CTG CTG GAT GCC TTT CTA CG | AGT TGT CCG ACA GGA AGC CGT A |
| Sstr2 | CAA GCA ATG GCT CCA ACC AGA C | CTT GGC ATA GCG GAG GAT GAC A |
| Sstr3 | CTT CGG ATC TCT CAT GTG CCG T | CAG ATA GCG GTC CAC ACT CAT G |
| Sstr4 | GCC ATC GGA TTA TGC TAC CTG C | CCA GCA TAG CAC AAA GAC GGT C |
| Sstr5 | TGG TCT TTG CGG ATG TCC AGG A | CAA AGA AGC CCA GCA CAG ACG T |
| Ppia | AGA CCA CAT GCT TGC CAT CCA G | CAT ACA GGT CCT GGC ATC TTG TC |
