# Supplementary material for: Beta cell primary cilia mediate somatostatin responsiveness via SSTR3
Source: Islets. 2023 Sep 3;15(1):2252855. doi: 10.1080/19382014.2023.2252855 (PMC10478741; doi:10.1080/19382014.2023.2252855)
Supplement: Supplemental Material [file KISL_A_2252855_SM3299.zip › Supplemental Table 2.docx]

Supplemental Table 2. Calcium Phenotype Summary

|  | Genetic knockout of primary cilia in beta cells | | shRNA knockdown of SSTR3 in islets | |
| --- | --- | --- | --- | --- |
|  | Wildtype (WT) | Beta cell cilia knockout (βCKO)  Ins1 Cre^+/+^ IFT88^fl/fl^ | Control | SSTR3 knockdown |
| SST applied after calcium oscillations induced by physiologic glucose | Calcium oscillations significantly suppressed after SST | Calcium oscillations continue and are Not suppressed after SST | Calcium oscillations significantly suppressed after SST | Calcium oscillations significantly suppressed after SST |
| SST and physiologic glucose applied simultaneously | Calcium oscillations are slowed | Calcium oscillations are Not slowed | Calcium oscillations are slowed | Calcium oscillations are Not slowed |
