# Supplementary figures and images for: Beta cell primary cilia mediate somatostatin responsiveness via SSTR3
Source: Islets. 2023 Sep 3;15(1):2252855. doi: 10.1080/19382014.2023.2252855 (PMC10478741; doi:10.1080/19382014.2023.2252855)

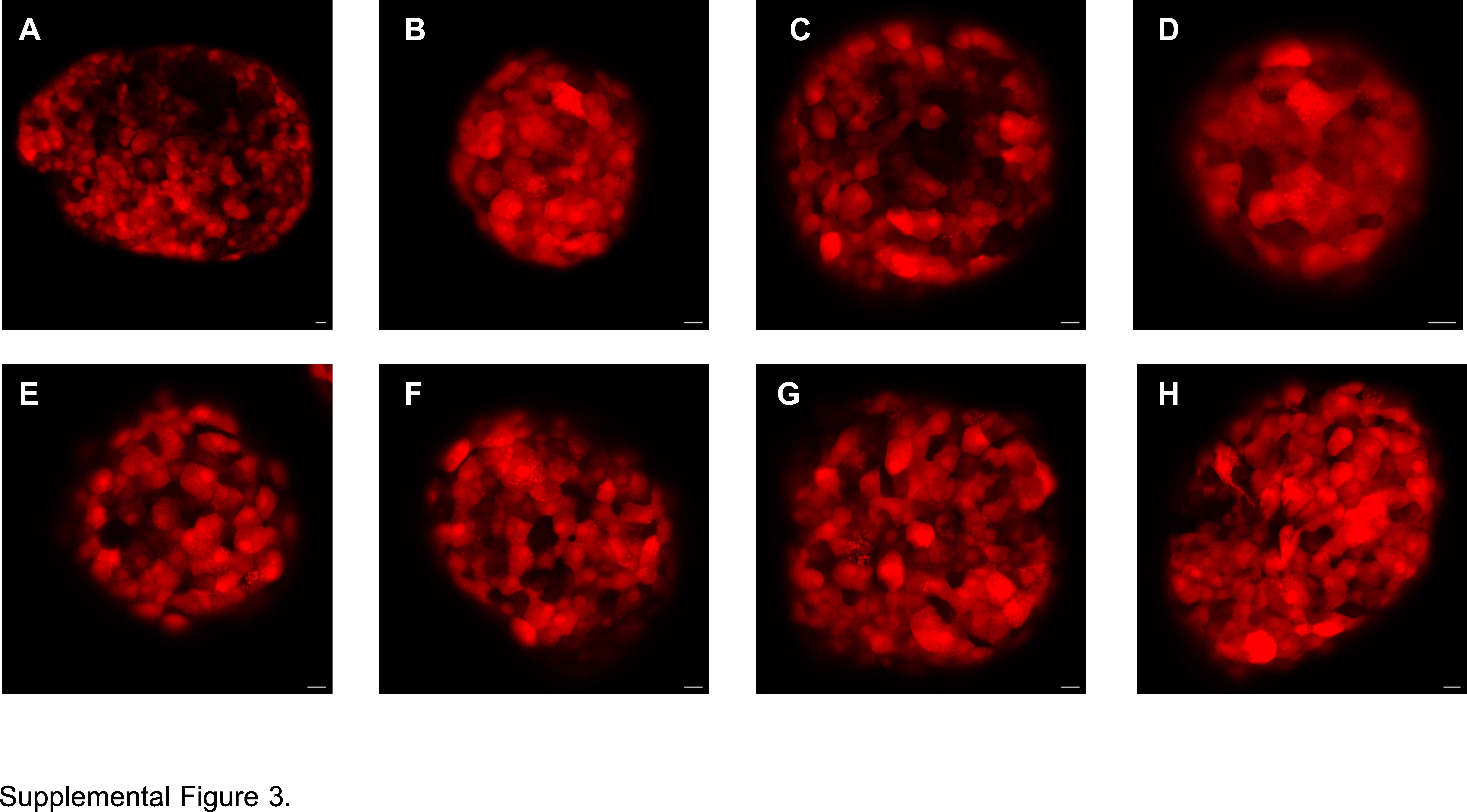

Supplement: Supplemental Material [file KISL_A_2252855_SM3299.zip › Supplemental Figure 3.jpg]
